# Supplementary material for: Epigallocatechin-3-Gallate Attenuates Myocardial Dysfunction via Inhibition of Endothelial-to-Mesenchymal Transition
Source: Antioxidants (Basel). 2023 May 7;12(5):1059. doi: 10.3390/antiox12051059 (PMC10215739; doi:10.3390/antiox12051059)

Supporting information

Supplementary Table S1. Primer sequences used for qRT-PCR

Primer name	Primer Sequence 5'→3'	Tm (℃)
COLIAI	Forward: ATGGATTCCCGTTCGAGTACG<br>Reverse: TCAGCTGGATAGCGACATCG	59
FSPI	Forward: TCAGGCAAAGAGGGTGACAAG<br>Reverse: AGGCAGCTCCCTGGTCAGT	60
SNAIL	Forward: GATGAGGACAGTGGCAAAAGCTC<br>Reverse: AGAATGGCTTCTCACCAGTGTGG	61
TGF βI	Forward: AGATTAAAATCAAGTGTGGAGCAAC<br>Reverse: GTCCTTCCTAAAGTCAATGTACAGC	59
VIMENTIN	Forward: GGTACAAGTCCAAGTTTGCTGACCT<br>Reverse: CATTGAGCAGATCTTGGTATTCACG	61
GAPDH	Forward: CATCACTGCCACCCAGAAGACTG<br>Reverse: ATGCCAGTGAGCTTCCCGTTCAG	60

Supplementary Figure S1. High-quality figures of Figure 4C

MI Snail

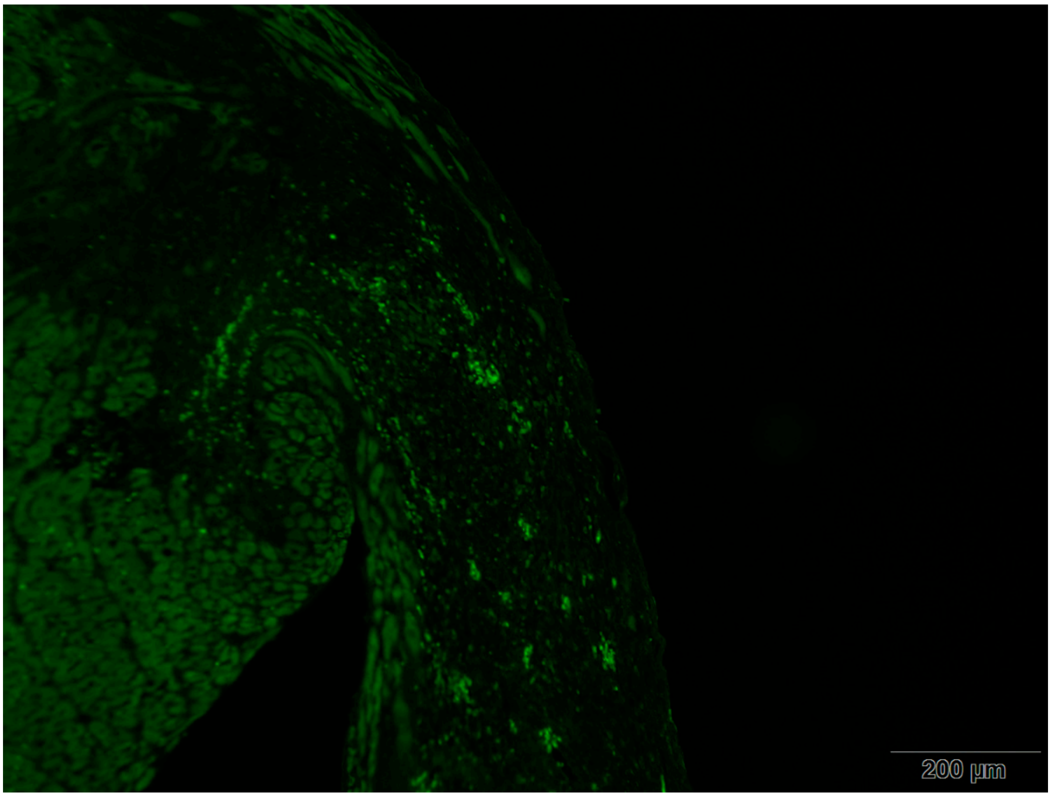

MI DAPI

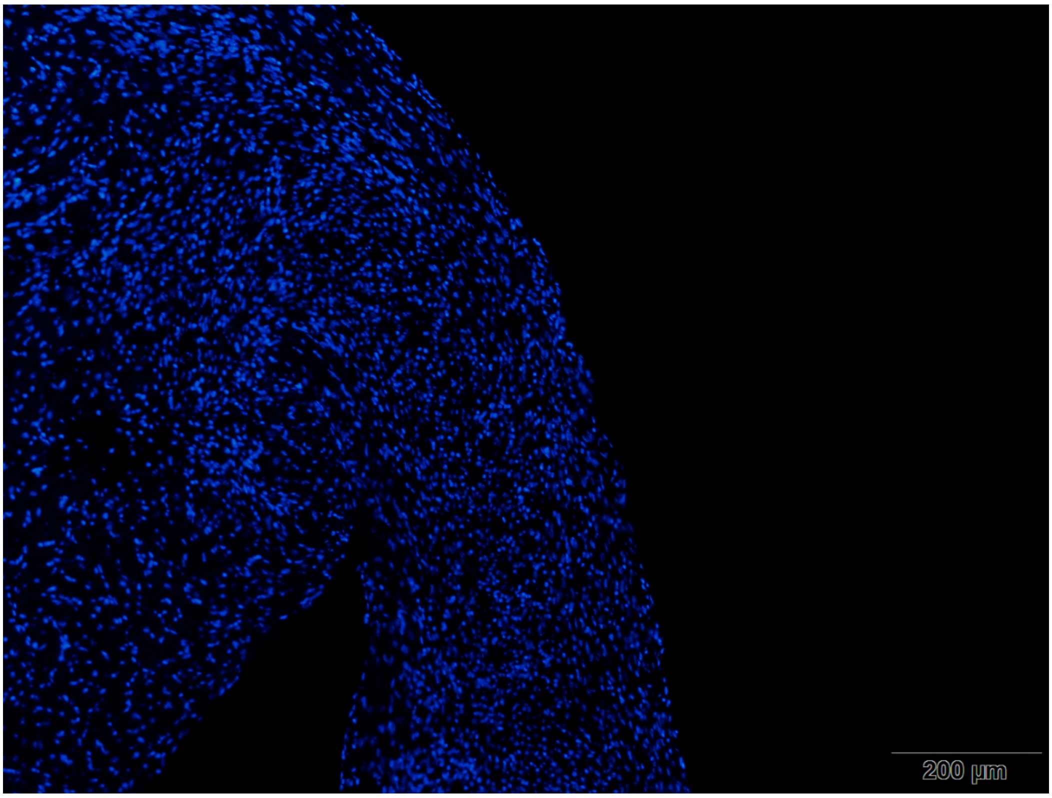

MI Merge

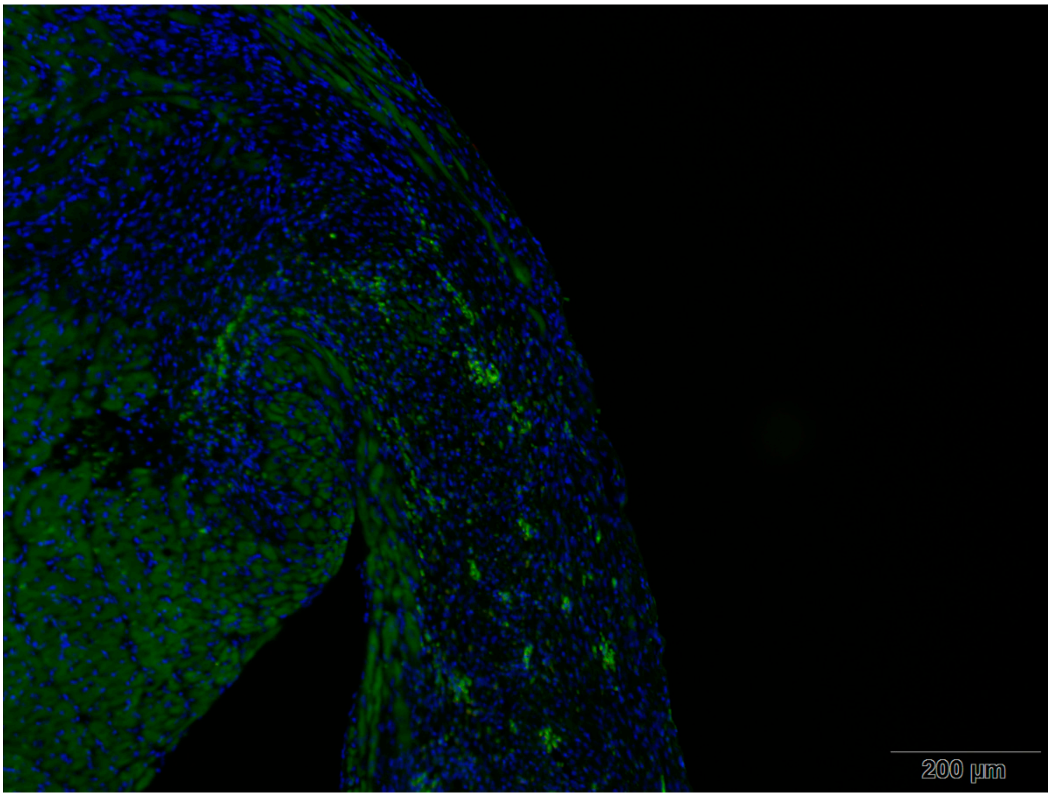

MI+EGCG Snail

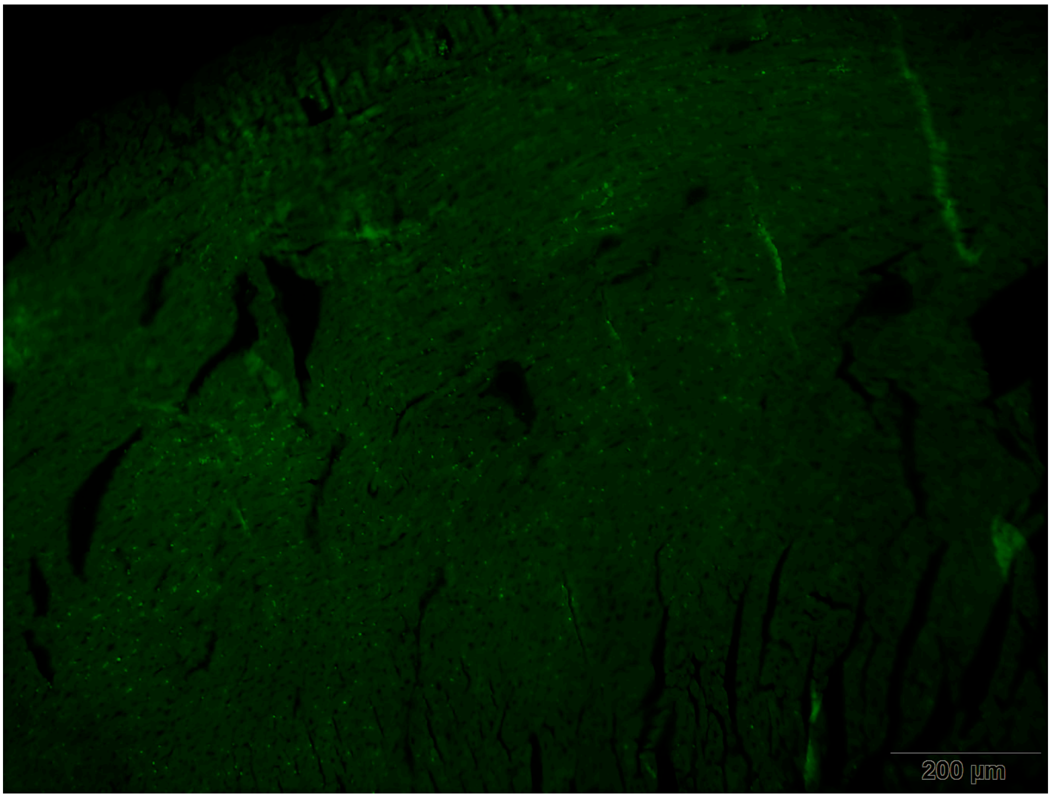

MI+EGCG DAPI

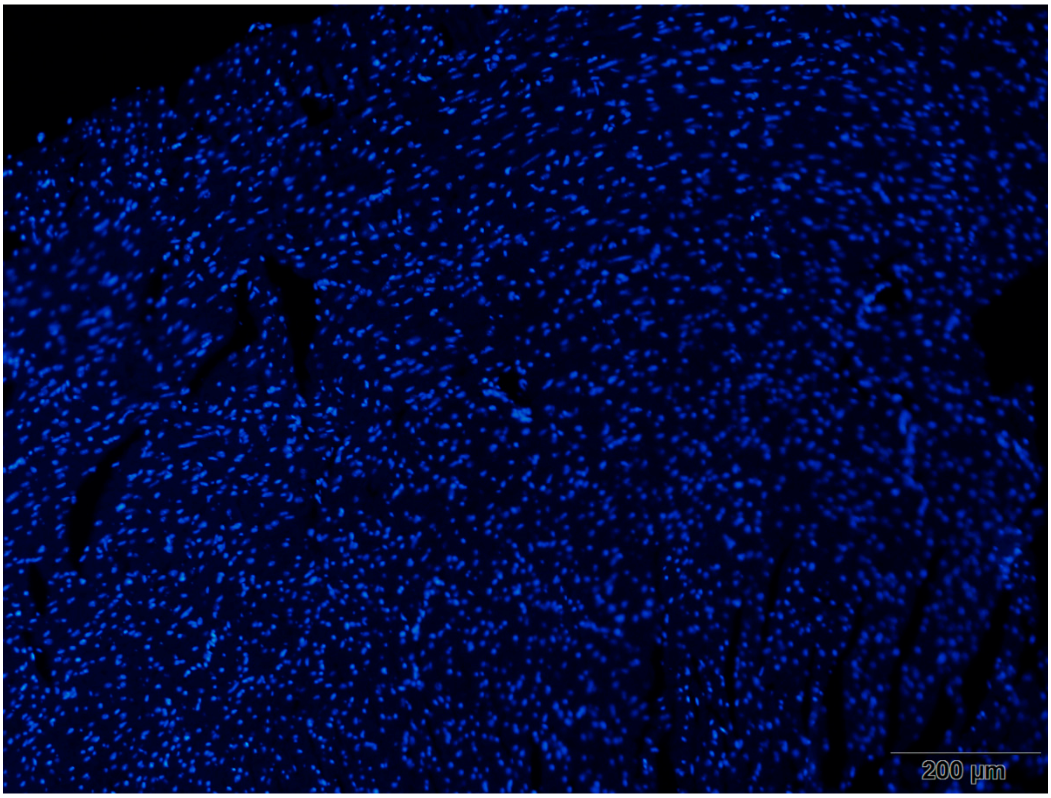

MI+EGCG Merge

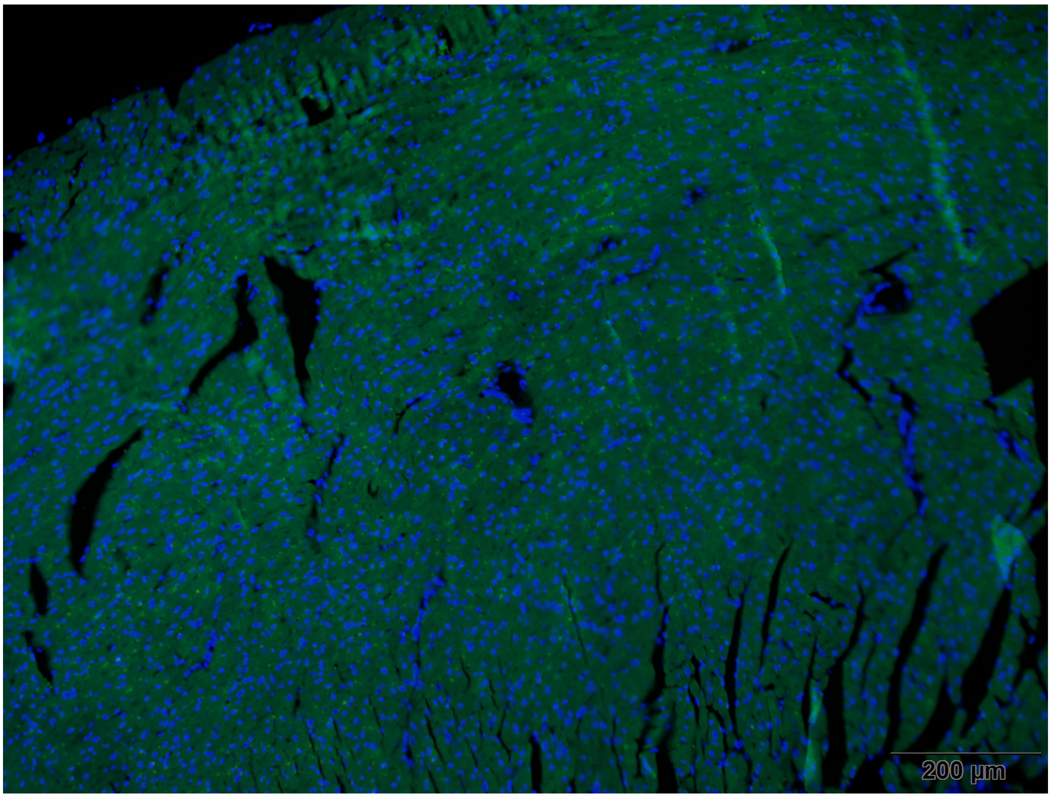

Supplementary Figure S2. High-quality figures of Figure 5B

MI 7-AAD

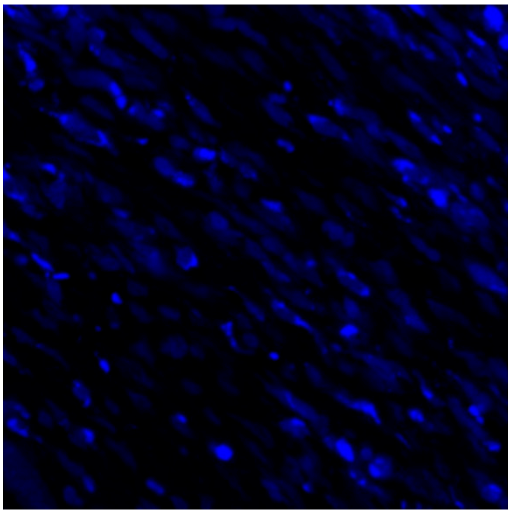

MI Br-dUTP

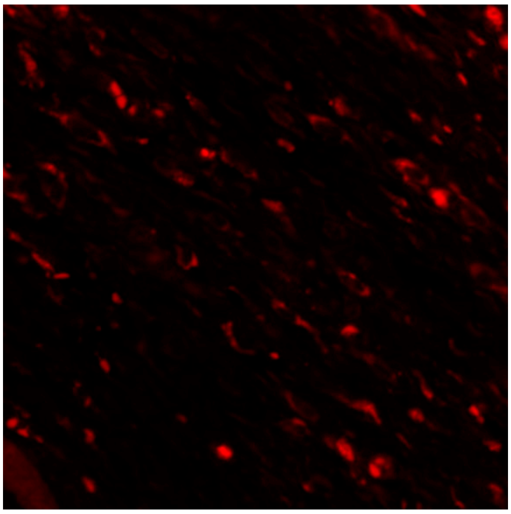

MI Merge

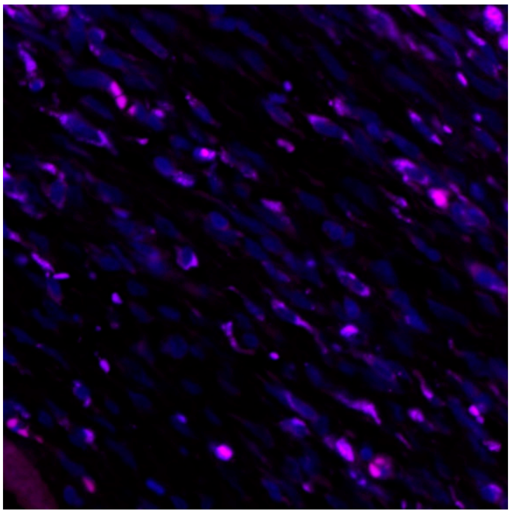

MI+EGCG 7-AAD

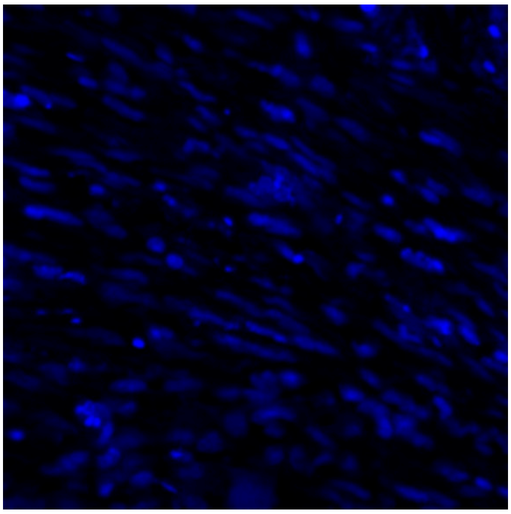

MI+EGCG Br-dUTP

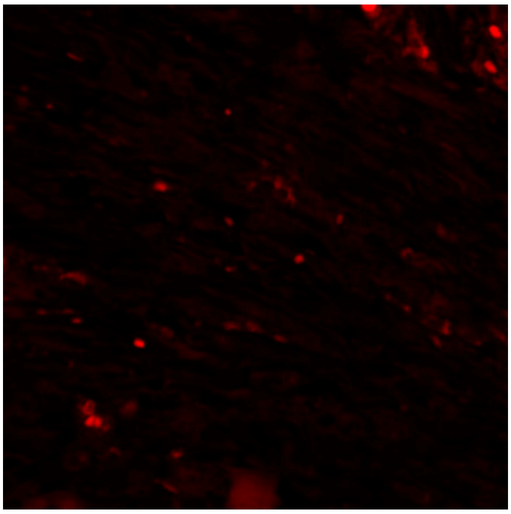

MI+EGCG Merge

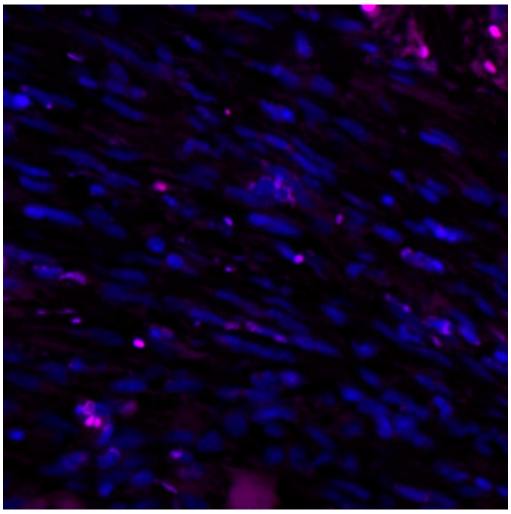

Supplementary Figure S3. High-quality figures of Figure 5D

MI CD31

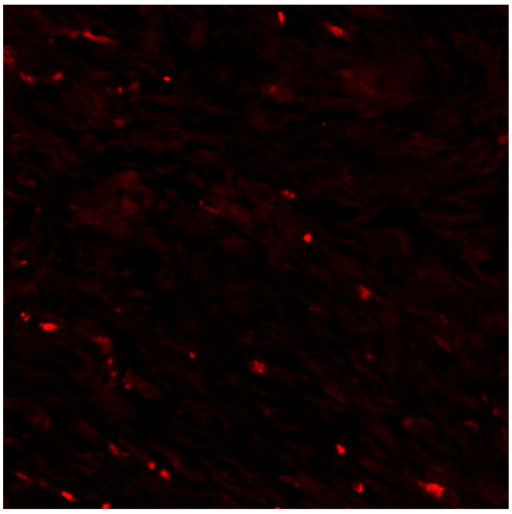

MI DAPI

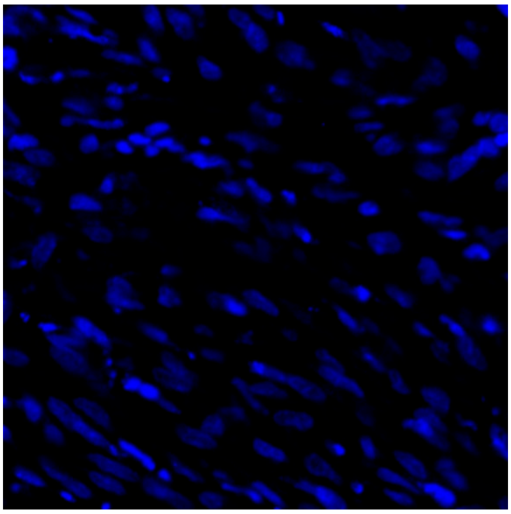

MI Merge

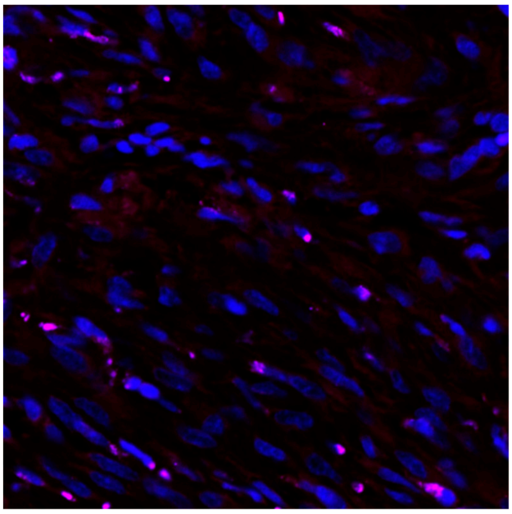

MI+EGCG CD31

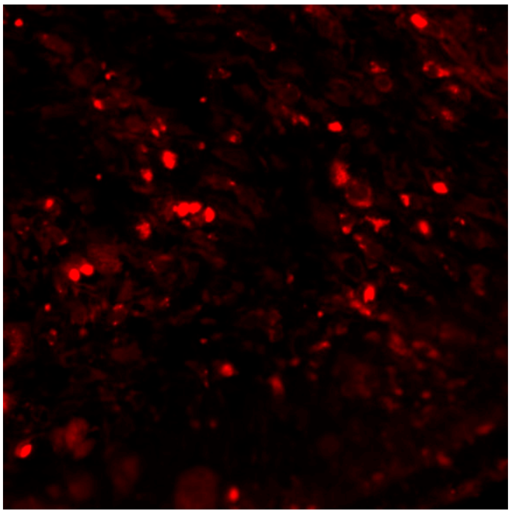

MI+EGCG DAPI

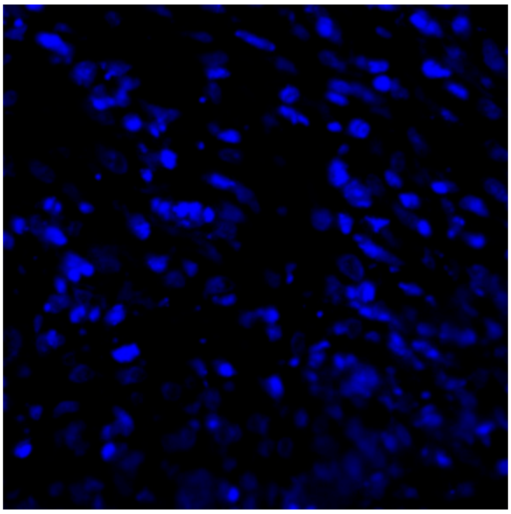

MI+EGCG Merge

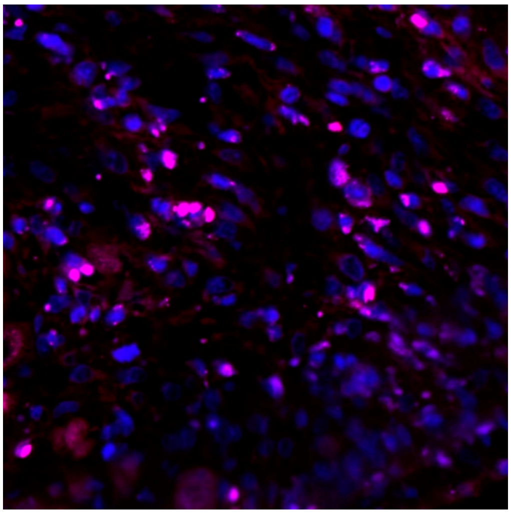

Supplement: Supplementary file 1 [file antioxidants-12-01059-s001.zip › antioxidants-2326733-supplementary.pdf]
